# Removal Efficiency of Sulfapyridine from Contaminated Surface Water by Carboxylated Graphene Oxide Blended PVDF Composite Ultrafiltration Membrane with Activated Carbon

**DOI:** 10.3390/polym14214779

**Published:** 2022-11-07

**Authors:** Yuliang Chen, Libo Ba, Yini He, Xuesong Yi

**Affiliations:** College of Ecology and Environment, Hainan University, Haikou 570228, China

**Keywords:** ultrafiltration membrane, carboxy-functionalized graphene oxide, sulfapyridine, antibiotics, response surface analysis

## Abstract

In this study, sulfapyridine (SPY), an antibiotic that is less commonly treated by membrane filtration techniques but is frequently detected in the aqueous environment and at higher concentrations than other detected antibiotics, was selected for investigation. A composite ultrafiltration membrane for the removal of sulfapyridine (SPY) antibiotics from water was fabricated using polyvinylidene fluoride (PVDF), polyvinylpyrrolidone (PVP), and carboxyl-functionalized graphene oxide (CFGO) as additives. The changes in retention rate and pure water flux of sulfapyridine by the composite ultrafiltration membrane were investigated by changing the ratios of the prepared ultrafiltration membrane materials under the conditions of low-pressure operation to explore the optimal experimental conditions. The results showed that the addition of PVP and CFGO significantly increased the number of membrane pores and their pore size. The addition of CFGO in the membrane significantly improved the hydrophilicity of the membrane. The contact angle decreased from 83.7 to 31.6°. Compared to ordinary PVDF ultrafiltration membranes, the membrane’s pure water flux increased nearly three times to 2612.95 L/(m^2^·h). The removal rate of SPY was 56.26% under the optimal conditions. When the composite ultrafiltration membrane was combined with activated carbon, the removal rate of SPY was 92.67%, which was nine times higher than that of activated carbon alone. At this time, the flux of the composite membrane was 2610.23 L/(m^2^·h). This study proposes a simple, efficient, and low production cost solution for the removal of sulfapyridine from water.

## 1. Introduction

Antibiotics are chemical substances secreted by microorganisms that have an inhibitory effect on the growth and reproduction of disease-causing microorganisms. Low doses of antibiotics discharged into the environment for long periods of time can cause increased resistance in sensitive bacteria, posing a potential threat to the ecological environment and human health [1,2,3]. Sulfonamide antibiotics (SAs) are p-aminobenzenesulfonamide-containing structural antibiotics, which are widely used in medicine, aquaculture, etc. Because humans and animals often cannot fully absorb antibiotics, many antibiotics enter water bodies as metabolites or even in their original form, causing pollution and forming micropolluted water [4]. Currently, the main methods for removing sulfonamide antibiotics from water are biological, physical, and chemical methods. Among them, treatment by biological methods may produce drug-resistant bacteria or superbugs, which can cause serious effects if the bacteria infect humans [5]. Ingerslev et al. found that microbial methods are less effective in degrading sulfonamide antibiotics [6]. Physical methods mainly include adsorption and membrane technology. The adsorption method has high treatment cost due to the high price of adsorbents. Adams et al. found that the effect of powdered activated carbon on Sulfonamide antibiotic (SA) removal was proportional to its dosing amount, but the study of sulfapyridine removal could not be carried out due to the problems of membrane contamination and activated carbon regeneration [7]. Membrane filtration alone can reduce the concentration of antibiotics in water to a certain extent, but it cannot remove them completely [8]. Chemical methods mainly include chlorination and advanced oxidation techniques. However, by-products from the treatment of sulfadoxine antibiotics by chlorination disinfection can cause secondary pollution of water bodies [9]. Therefore, the search for efficient treatment methods that do not cause secondary pollution to the environment has become an important issue in the current research field of antibiotic wastewater treatment.

Membrane treatment, especially reverse osmosis (RO) and nanofiltration (NF), can be suitable options in the search for the best solution for removal of sulfapyridine, a typical micropollutant organism in water [10]. However, the high operating pressure, low water flux, and high cost of NF and RO limit their large-scale application in water treatment [11]. Compared to NF and RO, UF has the advantages of high water flux, low operating pressure, and low cost [12]. Therefore, ultrafiltration has been widely used in water treatment to remove colloids, bacteria, and viruses [13]. Ultrafiltration membranes can partially remove microcontaminants from water. Despite the recent increase in interest in membrane technology for the removal of pharmaceutically active compounds [14,15], results for the removal of antibiotics are still scarce. In membrane technology, membrane filtration alone is able to reduce the concentration of sulfapyridine in water to some extent but not remove it completely [16]. Therefore, new additional data on the removal efficiency and rejection mechanisms of various membranes will help improve the understanding of antibiotic rejection. The removal of small molecule microcontaminants in the current ultrafiltration process has great potential for application, but it also presents a challenge for hybrid ultrafiltration membranes.

Based on these observations, the aim of this study was to produce a PVDF composite ultrafiltration membrane that can effectively remove sulfapyridine from water. This antibiotic was selected for study because it is less frequently treated by membrane filtration technology but is frequently detected in the aqueous environment and at higher concentrations than other antibiotics. Using this antibiotic as the target contaminant, the removal of the target antibiotic by PVDF ultrafiltration membrane technology was investigated by varying the PVDF content, PVP content, CFGO content, and PVP molecular weight in order to produce a PVDF composite ultrafiltration membrane with good removal effect on sulfapyridine. The adsorption rate of ordinary activated carbon for sulfapyridine is low at about 10% [17], and the cost of using modified activated carbon is high [18]. Therefore, in this experiment, activated carbon was used in combination with ultrafiltration membrane. After adding activated carbon to the original antibiotic solution, the antibiotic removal rate obtained by this method was tested using a CFGO composite ultrafiltration membrane. Finally, a solution with excellent removal performance for sulfapyridine was obtained.

## 2. Materials and Methods

### 2.1. Materials and Reagents

The carboxylated graphene oxide was obtained from a home-made laboratory in HaiKou, China. Polyvinylidene fluoride (PVDF) powder was available from China Sinopharm Group, Shanghai, China. Polyvinylpyrrolidone (PVP, 10,000 Da, AR) was supplied by Tianjin Damao Chemical Reagent Factory, China. Polyvinylpyrrolidone (PVP, 8000/58,000 Da, AR) and dimethylacetamide (DMAC, ≥98%) were supplied by Ron’s Reagent, Shanghai, China. Sulfapyridine (SPY, 100 μg/mL in methanol) was supplied by Source Leaf Reagent, Shanghai, China.

### 2.2. Preparation of Carboxyl-Functionalized Graphene Oxide

To obtain carboxyl-functionalized graphene oxide (CFGO), graphene oxide was added to distilled water, mixed with sodium hydroxide and bromoacetic acid (where GO:NaOH:C_2_H_3_BrO_2_ = 1:30:25), and placed in an ultrasonic water bath sonicator for 3 h (ultrasound frequency: 40 KHz, temperature: 30 °C). This process was carried out at room temperature in the laboratory. At the end of the shaking process, water and water-soluble components were removed from the solution, and the CFGO remaining in the solution was washed. Pure water was added to the mixture, centrifuged, and the supernatant was removed by centrifugation. Then, pure water was added again, centrifuged, and the supernatant was removed. This process was repeated seven more times while monitoring the pH of the mixture. After monitoring the pH of the mixture from 13 to about 7 for the first time, most of the impurities had been washed away, and the remaining mixture was dried in an oven at 60 °C to obtain solid CFGO.

### 2.3. Preparation of PVDF/CFGO Ultrafiltration Membrane

The PVDF/CFGO composite membrane was prepared by the classical submerged precipitation excited phase change method using DMAC as the solvent and PVP as the pore-forming agent. We obtained ultrafiltration membranes with CFGO contents of 0, 0.1, 0.2, 0.3, and 0.4%. An electronic balance in a beaker was used to weigh the corresponding PVDF, PVP, and DMAC (PVDF:PVP:DMAC = 16:4:80). Then, certain amounts of CFGO and PVP were stirred and dissolved in DMAC. The mixture was ultrasonically shaken for 5 min, mixed well, and the corresponding amount of PVDF was weighed. Then, PVDF was added, stirred well, and placed into an ultrasonicator with a lid to shake off the foam. The static solution was continuously stirred at room temperature for 1 h. After this, the paste solution obtained after shaking and defoaming the static solution was placed for 24 h to form a uniform cast film solution. The modified ultrafiltration membranes with different CFGO contents and 0.3 mm thickness were scraped out with a spatula and placed in air for 10–15 min. The naturally separated membranes were stored in deionized water for more than 12 h to stabilize and then stored with dropwise addition of formaldehyde to 0.5%.

### 2.4. BBD Response Surface Optimization Design

In this experiment, Box–Behnken design (BBD) combined optimization was performed using Design-Expert software (Version 10, Stat-Ease, Minneapolis, USA). BBD can optimize the experimental conditions of ultrafiltration membranes with the minimum number of experimental groups, and it can also study the interaction and relationship among the factors. PVDF content, PVP content, CFGO content, and PVP molecular weight were selected as variables, denoted as A, B, C, and D, respectively, and three levels were taken for each variation factor. In this way, the pure water flux and the removal rate of sulfapyridine by ultrafiltration membranes of 29 groups of PVDF were investigated. Details of the experimental design are shown in Table 1 and Table 2.

After the membrane material ratios with the best performance were experimentally obtained, the best ratios of ultrafiltration membranes were used for control experiments with a blank group with 19% PVDF content, 4% PVP content, 0% CFGO content, and 10,000 molecular weight PVP.

### 2.5. Determination of Pure Water Flux of Membranes

The pure water flux of CFGO-PVDF membranes was measured using an ultrafiltration cup device. The effective filtration area of the membrane was 50 cm^2^. The membrane was prepressurized at 25 °C and 0.05 MPa for 15 min. Then, distilled water was used as feed water, and the membrane was run for 30 min to stabilize. After a certain period of time, the pure water flow rate of the membrane was measured by the output volume. The formula for calculating the pure water flux is as follows:

$$J=\frac{V}{t\cdot S}$$

where *J* represents the pure water flux of the membrane in L/(m^2^∙h), *V* represents the water output in L, *t* denotes the operating time of the test instrument in h, and *S* denotes the effective filtration area of the membrane in m^2^.

### 2.6. Membrane Performance for Sulfapyridine Removal

Using an analytical balance, 20 mg of sulfapyridine was weighed and dissolved in 1000 mL of distilled water. A small portion of the water just after dissolving the antibiotic was taken as the original sample and the rest as the test sample. The antibiotic removal test was performed through an ultrafiltration cup, resulting in two cups of the treated antibiotic solution, 31 samples in total for the original sample, plus the test sample. The test membranes were placed in ultrafiltration cups at 25 °C under 0.05 MPa pressure and prepressurized with deionized water for 30 min. After running the instrument for a period of time, a certain amount of effluent was collected. This process is the conventional procedure for using the ultrafiltration cup, where one ultrafiltration membrane sample is tested at a time, then the next one is replaced, and the effluent is collected one by one. The removal rate was calculated by liquid chromatography.

The removal rate was calculated by the following formula:

$$R=\left(\frac{C_{p}-C_{f}}{C_{f}}\right)*100\%$$

where *R* denotes the retention rate of sulfapyridine by ultrafiltration membrane, *C_p_* denotes the sulfapyridine concentration of the effluent water, and *C_f_* denotes the sulfapyridine concentration of the influent water.

### 2.7. SPY Removal Rate of Ultrafiltration Membrane Combined with Activated Carbon

The obtained antibiotic solution was taken as it was, and graphene was added to it to make its concentration 1 g/L. After constant temperature oscillation for 2 h, the sample was obtained through the membrane, and the data obtained by liquid chromatography was compared with the original data to calculate the removal rate.

The calculation method and equation were the same as those outlined in Section 2.6.

## 3. Results and Discussion

### 3.1. Preparation of PVDF Composite Ultrafiltration Membrane and Response Surface Analysis

A series of CFGO-PVDF ultrafiltration membranes were prepared under different operating conditions according to the experimental grouping designed by BBD combination. In order to study the optimal ratio of CFGO-PVDF membrane, the pure water flux, sulfapyridine removal rate, and sulfapyridine removal rate after adding activated carbon of 29 composite ultrafiltration membranes were measured in this study. The results are shown in Table 3. From Table 3, it can be seen that the pure water flux range of CFGO-PVDF ultrafiltration membrane was 19–6900 L/(m^2^∙h), the sulfapyridine removal rate of CFGO-PVDF composite ultrafiltration membrane was 42.13–60.37%, and the sulfapyridine removal rate of CFGO-PVDF composite ultrafiltration membrane combined with activated carbon for sulfapyridine was 76.22–94.29%. The flux of pure water produced by the membranes varied under different conditions.

The results of pure water flux and sulfapyridine removal were obtained by testing 29 groups of membranes, and the data obtained were used for further examination and analysis.

#### 3.1.1. Significance Test

In the regression model using ANOVA, when the *p* value is less than 0.0500, it indicates that the effect of this influence factor is significant [19]. *p* = 0.0078 < 0.05 was used, with A representing PVDF content, B representing PVP content, C representing CFGO content, and D representing PVP molecular weight. As shown in Table 4, the model regression fit was good and significantly feasible. After ANOVA, the primary and secondary order of the effect of the four factors on pure water flux was A > D > B > C, i.e., PVDF content > PVP molecular weight > PVP content > CFGO content. Among them, the primary term A (PVDF content) and D (PVP molecular weight) had a great effect on the pure water flux of the composite ultrafiltration membrane (*p* < 0.01), the primary term B (PVP content) and the interaction term AC (PVDF and CFGO content) had a great effect on the results (*p* < 0.05), and the remaining terms had a small effect on the pure water flux of the composite ultrafiltration membrane.

#### 3.1.2. Response Surface Analysis

**Figure 1:**
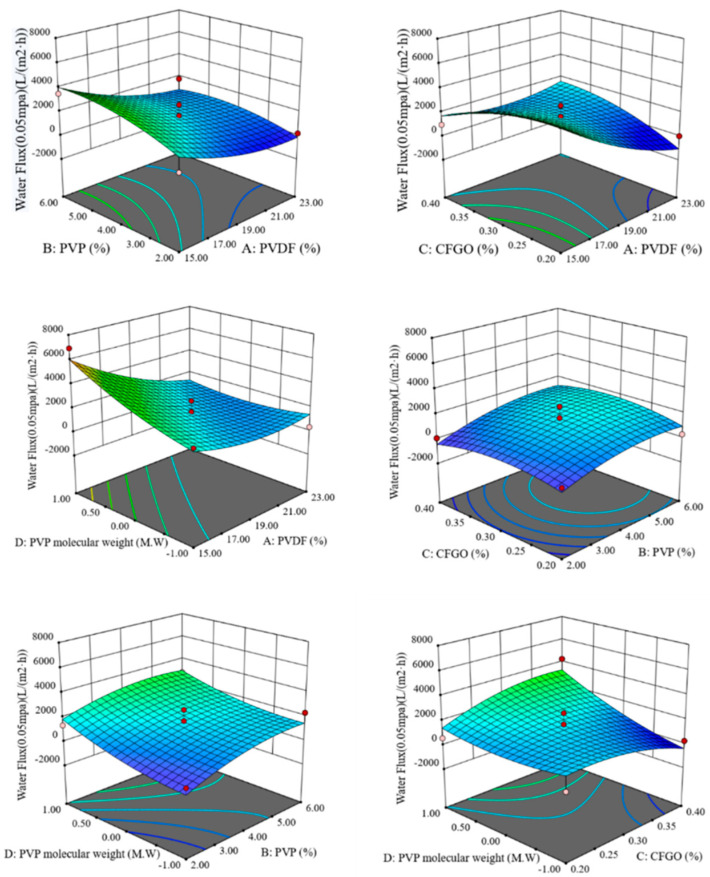
Response surface and contour of the interaction of various factors on pure water flux.

The response surface curves and contours of the interaction of PVDF content, PVP content, CFGO content, and PVP molecular weight on pure water flux are shown in Figure 1.

As can be seen from Figure 1, when the CFGO content and PVP molecular weight were constant, the pure water flux increased as the PVDF content decreased and the PVP content increased. When the PVP content and PVP molecular weight were constant, the pure water flux decreased with decreasing CFGO content when PVDF content was high and decreased with increasing CFGO content when PVDF content was low. When the PVP content and CFGO content were kept constant, the pure water flux increased as the PVDF content decreased and the molecular weight of PVP increased. When the PVDF content and PVP molecular weight were kept constant, the pure water flux only increased slightly with PVP content. When the PVDF content and CFGO content were fixed, the pure water flux increased with increasing PVP content and increasing PVP molecular weight. When the PVDF content and PVP content were fixed, the pure water flux increased with increasing PVP molecular weight when the CFGO content was higher. When the PVP molecular weight was larger, the pure water flux increased with the increase in CFGO content. When the PVP molecular weight was smaller, the pure water flux decreased with the increase in CFGO content.

The obtained data were fitted to obtain the regression equation:

Y = 1614.78 − 1353.87 × A + 729.68 × B + 39.18 × C + 941.43 × D − 261.94 × A × B + 1300.25 × A × C − 990.58 × A × D + 117.50 × B × C − 173.97 × B × D + 903.89 × C × D + 656.53 × A^2^ − 589.65 × B^2^ − 650.05 × C^2^ + 443.50 × D^2^ (1)

The maximum ratio of pure water flux was obtained by stepwise regression of the regression equation at 15% PVDF content, 5.34% PVP content, 0.28% CFGO content, and 58,000 molecular weight PVP, at which time the predicted value of pure water flux was 6313.63 L/(m^2^·h).

The *p*-values of the models for the remaining two data sets were less than 0.05, the *p*-values of the misfit term were greater than 0.05, and the reliability was high for both sets. The method given in Section 3.1.2 was used to calculate the remaining two sets of data.

The regression equation was obtained by fitting the data on the removal rate of sulfapyridine:

Y = 57.34 + 0.12 × A − 0.19 × B − 0.64 × C − 0.82 × D + 0.16 × A × B − 0.16 × A × C − 0.12 × A × D + 0.66 × B × C + 0.46 × B × D + 0.11 × C × D − 2.39 × A^2^ −2.26 × B^2^ − 7.15 × C^2^ − 3.06 × D^2^ (2)

The regression equation was obtained by fitting the data on the removal rate of sulfapyridine with the addition of activated carbon:

Y = 94.94 + 0.21 × A + 0.20 × B − 1.70 × C − 1.16 × D − 0.072 × A × B + 0.50 × A × C − 0.23 × A × D + 0.29 × B × C − 0.94 × B × D + 1.01 × C × D − 1.82 × A^2^ − 2.98 × B^2^ − 7.45 × C^2^ −2.96 × D^2^ (3)

The stepwise regression summary of the above three regression equations yielded the best ultrafiltration membrane ratio for the comprehensive data of 16.49% PVDF content, 4.41% PVP content, 0.27% CFGO content, and 9340 molecular weight PVP. Under this condition, the predicted value of pure water flux was 2653.31 L/(m^2^·h), the predicted value of sulfapyridine removal rate was 55.89%, and the predicted value of sulfapyridine removal rate with the addition of activated carbon was 93.86%. To facilitate the experiment and operation as well as the limitation of drugs, the optimal ultrafiltration membrane ratios were modified to 16.50% PVDF content, 4.50% PVP content, 0.30% CFGO content, and 10,000 molecular weight PVP. In this optimal condition, the pure water flux was 2612.95 L/(m^2^·h), the sulfapyridine removal rate was 56.26%, and sulfapyridine removal rate of sulfapyridine with the addition of activated carbon was 92.67%, which was more similar to the predicted value above, indicating the reliability of the three data models. This experiment focused on the performance of modified CFGO ultrafiltration membrane to remove sulfapyridine from water by comparing the original PVDF ultrafiltration membrane with the best performing CFGO composite ultrafiltration membrane. The main performance index parameters are shown in Table 5.

### 3.2. Morphology of CFGO/PVDF Ultrafiltration Membranes

**Figure 2:**
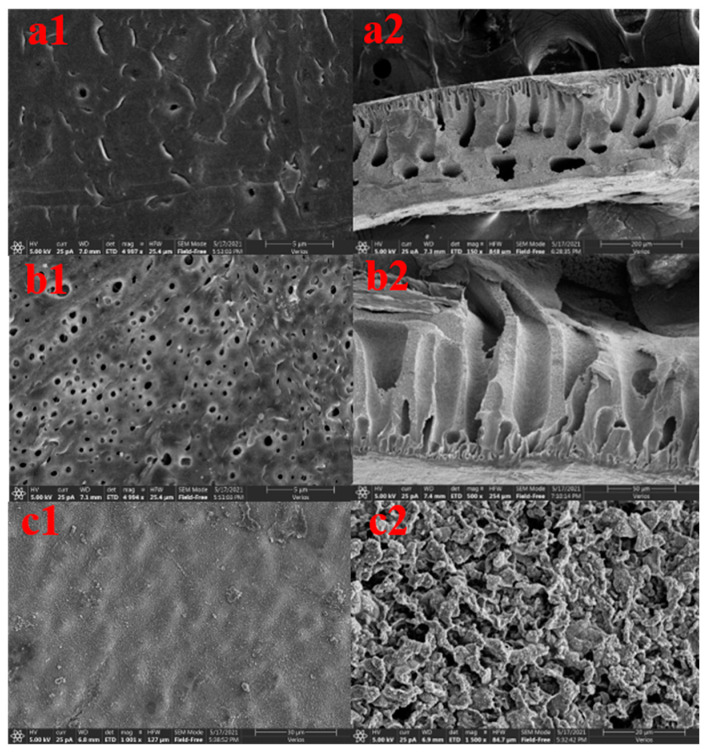
The CFGO ultrafiltration membrane with the best ratio and the ultrafiltration membrane with the worst removal performance of sulfapyridine at 5000× magnification. (**a1**,**b1**) SEM images of the lower surface; (**a2**,**b2**) SEM images of the cross section; and (**c1**,**c2**) SEM images of CFGO.

Figure 2a,b shows the microscopic morphology of the membrane surface of the ultrafiltration membrane with the worst sulfapyridine removal effect and the ultrafiltration membrane with the best sulfapyridine removal effect among the 29 groups of ultrafiltration membranes under scanning electron microscopy. The worst ultrafiltration membrane had fewer pores on the surface, which was consistent with the result of smaller water flux in the pure water flux measurement, while the best ultrafiltration membrane had more pores, which was consistent with the result of larger water flux in the pure water flux measurement. This is because the good hydrophilicity of CFGO accelerates the process of phase separation and makes the membrane pore channels larger. CFGO spontaneously moves up during phase separation and widens the membrane pore channels [20,21]. The addition of CFGO creates some lateral pores, and these lateral pores and enlarged finger pores increase the water flux of the membrane to some extent [22,23]. The variation in PVP content and PVP molecular weight is another factor. When PVP is used as a pore-forming agent, it will be enriched on the surface of the membrane. When the surface of the membrane is in contact with water, PVP will dissolve in water and form channels for the nonsolvent to enter the membrane’s interior. These points constitute the growth points of finger pores, which grow towards the membrane parent to form finger pores in the subsequent process [24,25]. The higher the PVP content, the higher the molecular weight, i.e., the higher the number of membrane pores.

### 3.3. Infrared Analysis of Membranes

**Figure 3:**
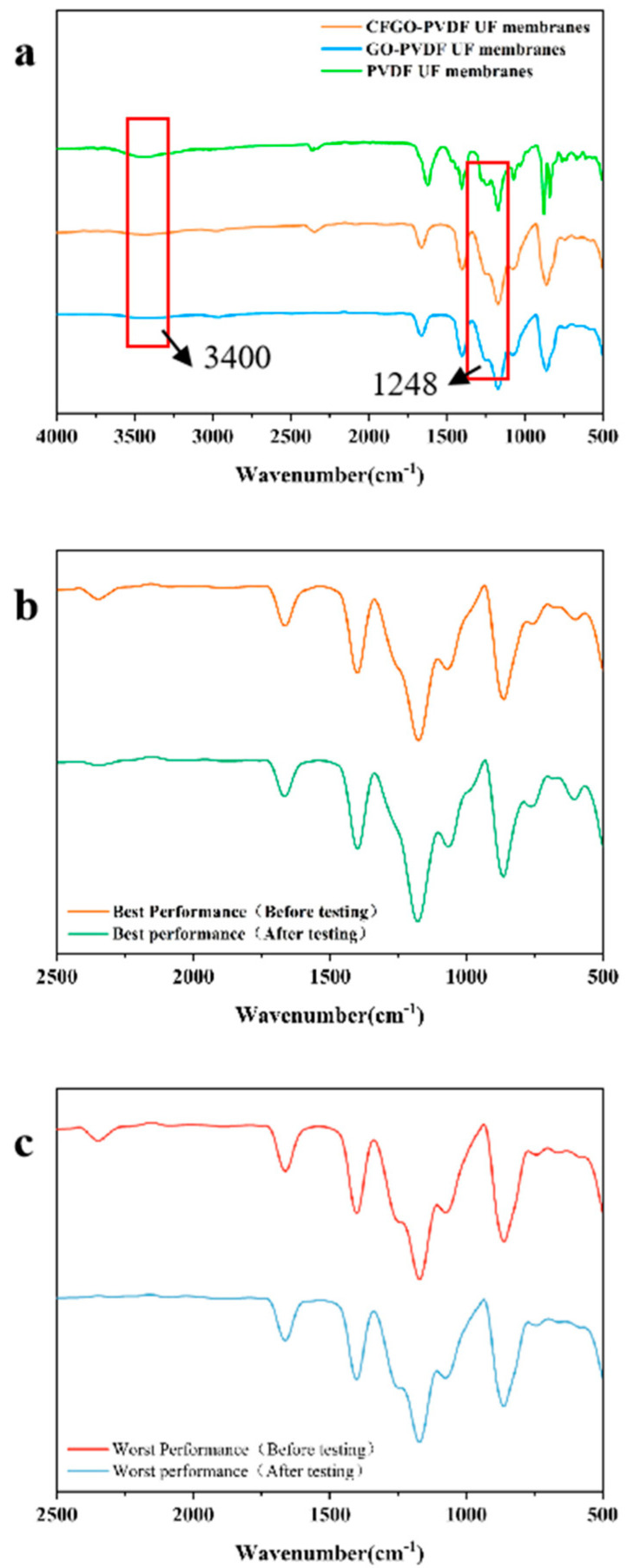
Infrared analysis of CFGO-PVDF ultrafiltration membranes. (**a**) The infrared spectra of CFGO co-blended PVDF ultrafiltration membranes with normal GO-PVDF ultrafiltration membranes and pure PCDF ultrafiltration membranes; (**b**) IR spectra of CFGO composite ultrafiltration membrane with the best sulfapyridine removal effect; and (**c**) IR spectra of CFGO composite ultrafiltration membrane with the worst sulfapyridine removal effect.

In order to investigate information relating to the functional groups on the surface of CFGO-PVDF ultrafiltration membrane, Fourier infrared (FTIR) analysis was performed on the composite membrane and its images were obtained in this study. Figure 3a represents the infrared spectra of CFGO co-blended PVDF ultrafiltration membrane and normal GO-PVDF ultrafiltration membrane. The characteristic absorption peaks of CFGO include the stretching vibration peaks of hydroxyl and carboxyl O-H bonds at 3400 cm^−1^, the stretching vibration peaks of C=C bonds at 1671 cm^−1^, C-H bonds at 3018 and 2978 cm^−1^, and the wave number structure of the stretching vibration peaks of epoxy C-O-C bonds at 1248.14 cm^−1^ [26,27]. The addition of CFGO significantly enhanced the characteristic peaks at 3400 cm^−1^ compared to the normal GO-PVDF films, and the increase in the content of these oxygen-containing functional groups significantly improved the hydrophilicity of the composite film surface. The absorption peaks of the stretching vibration of the --OH group (3400 cm^−1^) and the stretching vibration of the CO group (1599.72 cm^−1^) were both very strong, and the absorption peaks of the C-O-C group were greatly reduced, indicating that the carboxylated graphene oxide contained a large amount of carboxyl groups (-COOH), while the C-O-C group had been carboxylated.

Figure 3b shows the infrared spectra of the CFGO composite ultrafiltration membrane with the best removal of sulfapyridine, and Figure 3c shows the infrared spectra of the CFGO composite ultrafiltration membrane with the worst removal of sulfapyridine. From the graphs, it can be seen that no additional characteristic peaks appeared in the comparison of the ultrafiltration membrane before and after retention of the antibiotic sulfapyridine solution, indicating that the ultrafiltration membrane was not attached to sulfapyridine during the removal of the ultrafiltration membrane.

### 3.4. Contact Angle of Membrane Surface

In general, the hydrophilicity of organic membrane surface is usually expressed by the static contact angle of the surface, and the smaller the contact angle, the better the hydrophilicity of the membrane surface [28,29]. The contact angle of the ultrafiltration membrane without graphene was 83.66°, which was significantly larger than the contact angles for the other experimental groups, indicating that the addition of graphene could effectively reduce the contact angle of the ultrafiltration membrane. The contact angles of the ultrafiltration membranes in the experimental groups ranged from 31.6 to 65.8°, making them all hydrophilic ultrafiltration membranes. This was due to the hydrophilic group COOH in CFGO, which improves the hydrophilicity of PVDF films [30]. However, as the GO content continued to increase until the addition ratio was greater than 0.3 wt%, the contact angle became larger. This was probably due to the agglomeration of too many GO nanoparticles, which weakened the effective surface area of GO and reduced the amount of oxygen-containing functional groups exposed on the surface of GO [31], which in turn reduced the oxygen-containing functional groups on the surface of the composite membrane and in the membrane pores, thus leading to a larger contact angle. This suggests that only a low content of GO nanoparticles needs to be maintained in PVDF membranes in order to have better dispersion and more stable properties of the obtained composite membranes.

### 3.5. Membrane Performance Analysis

The CFGO-PVDF ultrafiltration membrane (16.50 wt% PVDF, 4.5 wt% PVP, 0.3 wt% CFGO, 10,000 molecular weight PVP) was selected as the optimal ratio for the test, and the composite ultrafiltration membrane (16.50 wt% PVDF, 4.5 wt% PVP, 0 wt% CFGO, 10,000 molecular weight PVP) was used as the control. Using high-pressure plate membrane small test equipment, the experiments of pure water flux and performance of sulfapyridine removal from water were conducted at room temperature. The ultrafiltration membrane was placed in an ultrafiltration cup, and nitrogen at a pressure of 0.05 MPa was used to test its permeation performance and desalination effect on actual seawater.

#### 3.5.1. Pure Water Flux of Membrane

**Figure 4:**
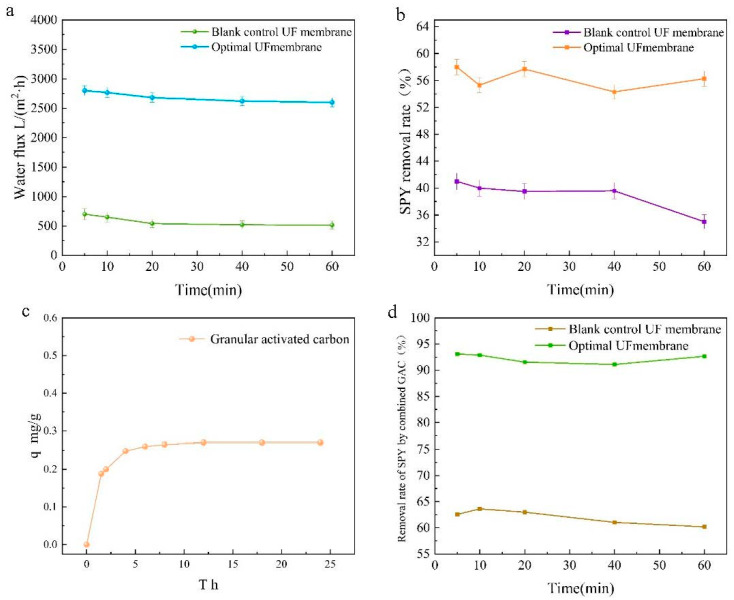
Performance test of CFGO-PVDF ultrafiltration membrane. (**a**) Pure water fluxes for the best ratio of CFGO-PVDF UF membrane and 0 wt% CFGO composite UF membrane; (**b**) removal performance of SPY from water with the best ratio of CFGO-PVDF UF membrane and 0 wt% CFGO composite UF membrane; (**c**) the kinetics of adsorption of sulfapyridine in water by activated carbon at room temperature (300 k); (**d**) the performance of CFGO-PVDF UF membrane and 0 wt% CFGO UF membrane in combination with AC for the removal of SPY in water.

**Figure 5:**
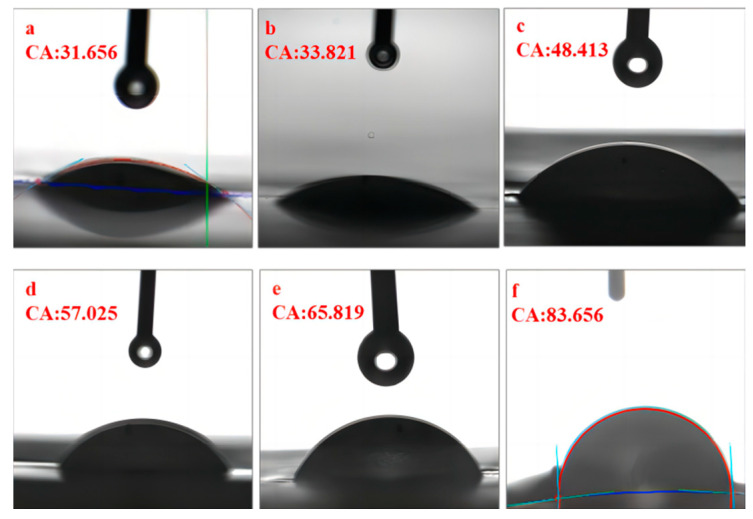
OCA analysis of six different CFGO-PVDF ultrafiltration membranes. (**a**–**e**) Composite UF membrane with minimum to maximum contact angle; (**f**) PVDFUF membrane without CFGO.

As shown in Figure 4a, pure water fluxes were tested for the best ratio of CFGO-PVDF ultrafiltration membrane and 0 wt% CFGO composite ultrafiltration membrane. The pure water fluxes of both membranes decreased at the beginning and stabilized after 30 min, but the total variation was small and the permeation stability was good. The CFGO composite ultrafiltration membrane with the best ratio achieved a pure water flux of 2612.95 L/(m^2^·h), which was surprisingly about four times higher than that of the 0 wt% CFGO composite ultrafiltration membrane. Combined with Figure 5, the contact angle promoted the hydrophilic performance of the composite membrane to different degrees with increasing CFGO nanoparticle addition, which was consistent with the change pattern of the pure water flux of the composite membrane. The pure water flux of ultrafiltration membranes with CFGO addition was significantly higher than that of PVDF membranes because the introduction of CFGO can increase the transport channels of water molecules, accelerate the permeation of water molecules, and enhance the hydrophilicity and permeability of the membrane [32]. The hydrophilic groups on the membrane surface can easily adsorb water molecules and form a hydration layer, which facilitates the preferential permeation of water through the membrane matrix.

#### 3.5.2. Analysis of Membrane Performance for Sulfapyridine Removal from Water

Figure 4b evaluates the removal performance of sulfapyridine from water with the best ratio of CFGO-PVDF ultrafiltration membrane and 0 wt% CFGO composite ultrafiltration membrane. The removal rate of sulfapyridine was 56.26% with the optimum ratio of CFGO-PVDF ultrafiltration membrane, and the removal performance fluctuated slightly with time and stabilized after 60 min. A total of 39.02% of sulfapyridine was removed by the 0 wt% CFGO composite ultrafiltration membrane, and the removal performance started to decrease after 20 min. This was due to the introduction of oxygen-containing functional groups on the surface of the optimally proportioned CFGO-PVDF ultrafiltration membrane and the high hydrophilicity of the membrane surface as well as the presence of acidic groups, such as carboxyl, phenolic hydroxyl, and carbon groups, on the surface of the CFGO composite ultrafiltration membrane, which can react with the basic amino groups carried by sulfapyridine and other sulfonamide antibiotics to form ionic bonds, thus enhancing the removal ability of sulfapyridine by the membrane [33].

Figure 4c shows the kinetics of adsorption of sulfapyridine in water by activated carbon at room temperature (300 k). The adsorption amount of activated carbon for sulfapyridine at adsorption equilibrium was q = 0.27 mg/g, and the removal rate of sulfapyridine by activated carbon was low at only 10.2%. This was due to the poor hydrophobicity of the activated carbon surface, which is a nonpolar adsorbent that has a low adsorption capacity for polar molecules dissolved in the aqueous phase [34].

To demonstrate an efficient and low-cost method for the removal of sulfapyridine from water as envisioned in this study, the composite ultrafiltration membranes were used in conjunction with activated carbon. Figure 4d presents the performance of CFGO-PVDF ultrafiltration membrane and 0 wt% CFGO ultrafiltration membrane in combination with activated carbon for the removal of sulfapyridine in water. The removal rate of sulfapyridine was 92.67% with the best ratio of CFGO-PVDF ultrafiltration membrane and activated carbon, and the removal performance fluctuated slightly with time and stabilized after 60 min. The removal rate of sulfapyridine was 60.65% with the 0 wt% CFGO composite ultrafiltration membrane and activated carbon, and the removal performance started to decrease after 20 min. The performance of the best CFGO-PVDF ultrafiltration membrane in combination with activated carbon for sulfapyridine removal was surprising, with a nearly 2-fold improvement compared to CFGO ultrafiltration membrane alone and a nearly 9-fold improvement compared to activated carbon alone. In this study, working at a pressure of 0.05 MPa, the same excellent results were achieved as Kosutic et al., who used nanofiltration and reverse osmosis membranes to remove sulfapyridine from water at high energy consumption [35], with significant cost and recovery savings compared to the use of modified activated carbon [36]. This proves that this study presents an efficient and low-cost solution for the removal of sulfapyridine from water.

## 4. Conclusions

A series of CFGO-PVDF composite ultrafiltration membranes were fabricated by co-blending carboxyl-functionalized graphene oxide (CFGO) with PVDF. The optimal ratios were found by BBD response surface optimization design experiments. Comparing the permeation performance and sulfapyridine removal performance of CFGO composite ultrafiltration membranes with different ratios, the best ultrafiltration membrane ratio was determined by the response surface design to be 16.50% PVDF content, 4.50% PVP content, 0.30% CFGO content, and 10,000 molecular weight PVP. In this optimal condition, the pure water flux was 2612.95 L/(m^2^·h), the removal rate of sulfapyridine was 56.26%, and the removal rate of sulfapyridine in combination with activated carbon was 92.67%. The pure water flux was three times higher than that of the ordinary PVDF ultrafiltration membrane, which had good permeability and hydrophilicity. When the CFGO composite ultrafiltration membrane was used in combination with activated carbon, it produced a surprising effect, with the 92.67% sulfapyridine removal rate far exceeding the removal performance of the ultrafiltration membrane or activated carbon working alone. The problems of low removal rate of sulfapyridine antibiotics by unmodified activated carbon, incomplete removal of sulfapyridine antibiotics from water by ultrafiltration membranes, and high energy consumption required by nanofiltration or reverse osmosis membranes were solved. The results show that this study has come up with a simple, efficient, and low production cost method for the removal of sulfapyridine in water, which provides a very valuable solution to the present problem of commonly detected sulfapyridine antibiotics in water.

## Figures and Tables

**Table 1:** Influencing factors and levels of experimental design.

Level	PVDF Content%	PVP Content%	CFGO Content%	PVP Molecular Weight—
**−** **1**	15	2	0.2	8000
**0**	19	4	0.3	10,000
**1**	23	6	0.4	58,000

**Table 2:** BBD response surface design of experiments.

Std	A:PVDF	B:PVP	C:CFGO	D:PVP Molecular Weight
	%	%	%	—
**1**	15	2	0.3	10,000
**2**	23	2	0.3	10,000
**3**	15	6	0.3	10,000
**4**	23	6	0.3	10,000
**5**	19	4	0.2	8000
**6**	19	4	0.4	8000
**7**	19	4	0.2	58,000
**8**	19	4	0.4	58,000
**9**	15	4	0.3	8000
**10**	23	4	0.3	8000
**11**	15	4	0.3	58,000
**12**	23	4	0.3	58,000
**13**	19	2	0.2	10,000
**14**	19	6	0.2	10,000
**15**	19	2	0.4	10,000
**16**	19	6	0.4	10,000
**17**	15	4	0.2	10,000
**18**	23	4	0.2	10,000
**19**	15	4	0.4	10,000
**20**	23	4	0.4	10,000
**21**	19	2	0.3	8000
**22**	19	6	0.3	8000
**23**	19	2	0.3	58,000
**24**	19	6	0.3	58,000
**25**	19	4	0.3	10,000
**26**	19	4	0.3	10,000
**27**	19	4	0.3	10,000
**28**	19	4	0.3	10,000
**29**	19	4	0.3	10,000

**Table 3:** Box–Behnken test results.

Number	Water Flux (0.05 mpa) L/(m^2^∙h)	Sulfapyridine Removal Rate	Sulfapyridine Removal Rate after Adding Activated Carbon
1	865.12	44.53%	82.22%
2	185.95	45.47%	81.05%
3	3501.82	44.99%	84.82%
4	1774.88	46.59%	77.61%
5	146.45	47.80%	86.46%
6	239.67	42.13%	83.99%
7	569.59	46.31%	83.52%
8	4278.35	46.09%	86.79%
9	2381.65	53.13%	78.22%
10	330.08	51.57%	78.83%
11	6922.31	45.00%	85.80%
12	908.43	46.98%	85.95%
13	48.26	50.87%	79.02%
14	283.47	47.60%	88.21%
15	73.31	52.51%	80.04%
16	778.51	46.87%	88.81%
17	5507.11	49.20%	83.79%
18	19.17	47.75%	90.91%
19	980.66	58.15%	78.94%
20	693.72	51.06%	84.63%
21	146.94	48.59%	86.59%
22	2289.92	52.71%	83.71%
23	1353.06	47.39%	76.22%
24	2800.17	60.37%	83.84%
25	860.5	57.59%	91.89%
26	1613.22	56.96%	89.69%
27	1348.76	56.65%	94.29%
28	2584.96	58.82%	88.76%
29	1666.44	56.66%	89.49%

**Table 4:** Analysis of variance of pure water flux response surface test results.

Source of Variance	Sum of squares	Degree of Freedom	Mean Square	F-Value	*p*-Value	Influence Degree
Models	6.46	14.00	4.62	3.91	0.0078	**
A-PVDF	2.20	1.00	2.20	18.62	0.0007	**
B-PVP	6.39	1.00	6.39	5.41	0.0356	*
C-CFGO	1.84	1.00	1.84	0.02	0.9024	
D-PVP molecular weight	1.06	1.00	1.06	9.00	0.0095	**
AB	2.74	1.00	2.74	0.23	0.6373	
AC	6.76	1.00	6.76	5.72	0.0313	*
AD	3.92	1.00	3.92	3.32	0.0898	
BC	5.52	1.00	5.52	0.05	0.8320	
BD	1.21	1.00	1.21	0.10	0.7536	
CD	3.27	1.00	3.27	2.77	0.1185	
A^2^	2.80	1.00	2.80	2.37	0.1463	
B^2^	2.26	1.00	2.26	1.91	0.1887	
C^2^	2.74	1.00	2.74	2.32	0.1500	
D^2^	1.28	1.00	1.28	1.08	0.3163	
Residuals	1.65	14.00	1.18			
Loss of proposed items	1.50	10.00	1.50	3.78	0.1060	
Error term	1.58	4.00	3.96			
Total	8.12	28.00				

Note: * indicates that the item had a large effect on the results (*p* < 0.05); ** indicates that the item had a large effect on the results (*p* < 0.01).

**Table 5:** The main index parameters of ultrafiltration membrane.

Parameters	PVDF UF Membrane	CFGO UF Membrane
Surface thickness (mm)	0.3	0.3
Main Materials	PVDF	PVDF, CFGO Pellets
Average pore size(nm)	8–10	8–16
Contact angle (°)	83.7	31.6
Cutting molecular weight (Da)	100,000 Da	100,000 Da
Pure water flux		
L/(m^2^·h)	980	2912

Note: The pure water flux here was obtained by measuring the volume of deionized water passing through the diaphragm at a certain time at a pressure of 0.05 MPa and by calculation.

## Data Availability

The corresponding author does not have permission to submit the data in this study.
